# Impact of COVID-19 pandemic and lockdown on eye emergencies

**DOI:** 10.1177/1120672120974944

**Published:** 2020-11-19

**Authors:** Alicia Poyser, Sundeep S Deol, Lina Osman, Helen J Kuht, Tharsica Sivagnanasithiyar, Roslyn Manrique, Linda O Okafor, Ian DeSilva, David Sharpe, Vijay Savant, Usman Sarodia, Nagini Sarvananthan, Ray Chaudhuri, Somnath Banerjee, Joyce Burns, Mervyn G Thomas

**Affiliations:** 1 Emergency eye services, Department of Ophthalmology, University Hospitals of Leicester, Leicester Royal Infirmary, Leicester, UK; 2 The University of Leicester Ulverscroft Eye Unit, Department of Neuroscience, Psychology and Behaviour, University of Leicester, RKCSB, Leicester, UK; 3 Vitreoretinal service, Department of Ophthalmology, University Hospitals of Leicester, Leicester Royal Infirmary, Leicester, UK; 4 Oculoplastics service, Department of Ophthalmology, University Hospitals of Leicester, Leicester Royal Infirmary, Leicester, UK; 5 IT services, University Hospitals of Leicester, Leicester Royal Infirmary, Leicester, UK; 6 Corneal service, Department of Ophthalmology, University Hospitals of Leicester, Leicester Royal Infirmary, Leicester, UK; 7 Glaucoma service, Department of Ophthalmology, University Hospitals of Leicester, Leicester Royal Infirmary, Leicester, UK; 8 Neuro-ophthalmology and Paediatric Ophthalmology service, Department of Ophthalmology, University Hospitals of Leicester, Leicester Royal Infirmary, Leicester, UK

**Keywords:** COVID-19, pandemic, retinal detachment, lockdown, emergency

## Abstract

**Background:**

To characterise and compare ocular pathologies presenting to an emergency eye department (EED) during the COVID-19 pandemic in 2020 against an equivalent period in 2019.

**Methods:**

Electronic patient records of 852 patients in 2020 and 1818 patients in 2019, attending the EED at a tertiary eye centre (University Hospitals of Leicester, UK) were analysed. Data was extracted over a 31-day period during: (study period 1 (SP1)) COVID-19 pandemic lockdown in UK (24th March 2020–23rd April 2020) and (study period 2 (SP2)) the equivalent 2019 period (24th March 2019–23rd April 2019).

**Results:**

A 53% reduction in EED attendance was noted during lockdown. The top three pathologies accounting for >30% of the caseload were trauma-related, keratitis and uveitis in SP1 in comparison to conjunctivitis, trauma-related and blepharitis in SP2. The overall number of retinal tears and retinal detachments (RD) were lower in SP1, the proportion of macula-off RD’s (84.6%) was significantly (*p* = 0.0099) higher in SP1 (vs 42.9% in SP2).

**Conclusion:**

COVID-19 pandemic related lockdown has had a significant impact on the range of presenting conditions to the EED. Measures to stop spread of COVID-19 such as awareness of hand hygiene practices, social distancing measures and school closures could have an indirect role in reducing spread of infective conjunctivitis. The higher proportion of macula-off RD and lower number of retinal tears raises possibility of delayed presentation in these cases. Going forward, we anticipate additional pressures on EED and other subspecialty services due to complications and associated morbidity from delayed presentations.

## Introduction

COVID-19 is caused by a severe acute respiratory syndrome coronavirus 2 (SARS-CoV-2) infection and was declared a pandemic by the World Health Organisation (WHO) on 11th March 2020.^1^ As of 2nd August 2020, more than 17 million cases of COVID-19 with over 680,000 deaths have been reported worldwide.^2^ Similarly, in the United Kingdom (UK), over 300,000 COVID-19 confirmed cases and in excess of 46,000 related deaths have been reported.^2^ The first case of COVID-19 was reported in the UK on 30th January 2020, and the first mortality on 5th March 2020.^3^ In response to this pandemic, the UK government issued national lockdown orders on the 23rd March 2020, with an aim to contain the rates of infection and thus reduce anticipated pressures on the National Health Service (NHS). Moreover, additional restrictions such as strict home isolation was advised for ‘clinically vulnerable’ (e.g. those aged 70 years or older) and ‘clinically extremely vulnerable’ individuals (e.g. patients on immunosuppression therapies).^4^ The Royal College of Ophthalmologists (RCOphth), UK, have provided guidance on ophthalmic service delivery during the COVID-19 pandemic. This includes switching to virtual or telephone consultations and deferring elective procedures such as cataract surgery where possible.^5^ In the United States, data from the National Patient and Procedure Volume Tracker^TM^ reports a reduction in volume of patients across most specialities, with ophthalmology being most severely affected (81% drop in patient volume).^6^ Across specialities, high volume patient visits and procedures that dropped the most were for cataracts (97%).^6^ However, emergency eye services at NHS trusts have continued to provide face-to-face consultations and emergency eye care, albeit with limited staff due to re-deployment.^7^ The effects of these measures on individuals with ocular pathologies are unclear.

Pandemics can influence patient behaviour in seeking emergency medical treatment.^8^ Data from the Emergency Department Syndromic Surveillance System: England (EDSSS) and the Royal College of Emergency Medicine, UK, showed that attendances to the emergency department were down by 25% to 50% during first week of lockdown in the UK.^9^ This raises the possibility that some people may be harmed by not accessing appropriate treatment in a timely manner. There is increasing concern that patients with life threatening disorders (such as myocardial ischaemia) are not seeking medical help appropriately.^9^ It is unclear whether a similar trend is seen amongst sight threatening disorders and specifically the impact of the lockdown on eye emergencies. We aimed to characterise and compare ocular pathologies presenting to a busy tertiary emergency eye department in the UK.

## Methods

Electronic patient records (EPR) of all patients attending the emergency eye department (EED) at a tertiary eye centre (University Hospitals of Leicester (UHL), UK) were analysed.

The EED at UHL receives patients from opticians, primary care physicians and ‘walk-in’ individuals. Due to expertise in all ophthalmic sub-specialities, EED at UHL is a tertiary referral centre covering a large geographic area and a diverse population.

UHL uses an integrated EPR system, Nervecentre (Nervecentre Software Ltd, Wokingham UK), which holds all patient demographic and clinical data related to EED attendance. We defined two, 31-day study periods to allow for comparisons between 2020 and 2019. Data was extracted over a 31-day period during: (study period 1 (SP1)) the COVID-19 pandemic lockdown in UK (24th March 2020–23rd April 2020) and (study period 2 (SP2)) the same period during 2019 (24th March 2019–23rd April 2019).

From EPR we extracted the number of patients attending EED, patient demographic data (age and gender) and diagnoses. Additional data from procedure logbooks (theatre and laser) and the local Picture Archiving and Communication System (PACS) servers were obtained to corroborate findings.

This project was implemented by the EED core study group (AP, SSD, LO, TS, RM, LOO, JB and MGT) with full support from each of the clinical subspecialty and UHL audit teams. In accordance with local UHL institutional policy, the project was reviewed and registered (registration number: 10568).

To compare differences in proportion of cases of different diagnoses between SP1 and SP2, we performed a Chi-squared test or a Fisher’s exact test (when *E* < 5; where *E* is the expected frequency). To compare differences in age between SP1 and SP2 we used an unpaired *t*-test (data was normally distributed with equal variances). All analyses were considered significant at a type 1 probability value of *p* < 0.05. Statistical analysis was performed with IBM^®^ SPSS^®^ Statistics v26.0 (SPSS, Inc., Chicago, IL).

## Results

### Overview

**Figure 1.**
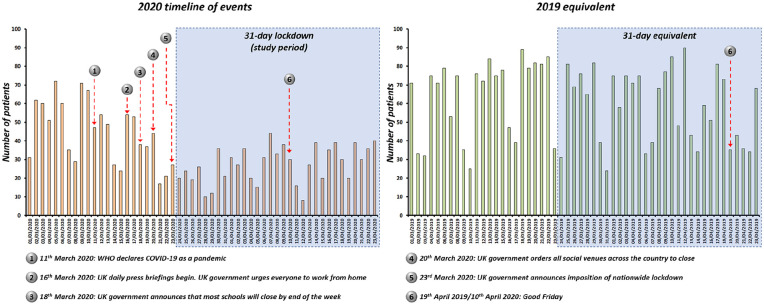
Timeline of events preceding and during COVID-19 related pandemic lockdown in the UK. Bar chart shows number of patients presenting to the eye emergency department (EED) at in 2020 and an equivalent period in 2019. The study period represents a 31-day lockdown period. Predictable drop in EED is seen during weekends and public holidays (e.g. Good Friday) in 2019. In 2020, we observe a decline in EED attendance as government escalates social distancing measures and imposes nationwide lockdown.

The timeline of events in relation to the number of EED attendances are shown in Figure 1 for the study period 1 and 2. In the month of March 2020, there is a gradual decline in the number of patients attending EED as the UK government escalates social distancing measures. After the announcement of the nationwide lockdown (23rd March 2020), we observe the lowest numbers of patients attending EED during SP1 (Figure 1). Contrastingly, during SP2, high levels of EED attendances are noted with predictable dips during weekends and public holidays (e.g. Good Friday 19th April 2019).

**Figure 2.**
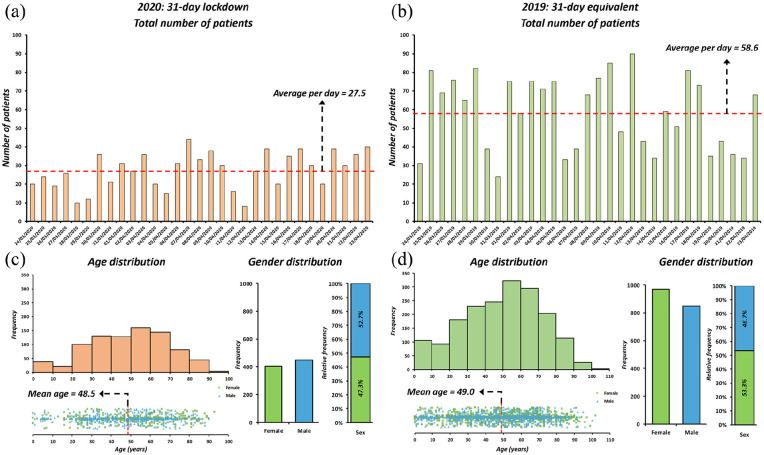
(a) Bar chart of eye emergency department during 31-day study period with average number of attendances per day during COVID-19 related pandemic lockdown, (b) compared to the equivalent period in 2019, (c) age and gender distribution for the same period during 2020, and (d) 2019 are also shown.

Over the study periods there was a 53.1% reduction in EED attenders between SP1 (*n* = 852) and SP2 (*n* = 1818). The average number of attendances per day in SP1 were 27.5 (SD = 9.6), while during SP2 it was 58.6 (SD = 19.4) (Figure 2).

There was no significant difference (*p* > 0.05) between the ages of patients presenting to EED during SP1 (mean ± SD = 48.5 ± 22.1 years) and SP2 (mean ± SD = 48.5 ± 22.1 years). During SP1 we observed a significantly (*p* = 0.0038) higher proportion of males (52.7%) and lower proportion of females (47.3%) attending EED compared to SP2 (males = 46.7%, females = 53.3%) (Figure 2).

### Trends in top diagnostic categories

**Figure 3.**
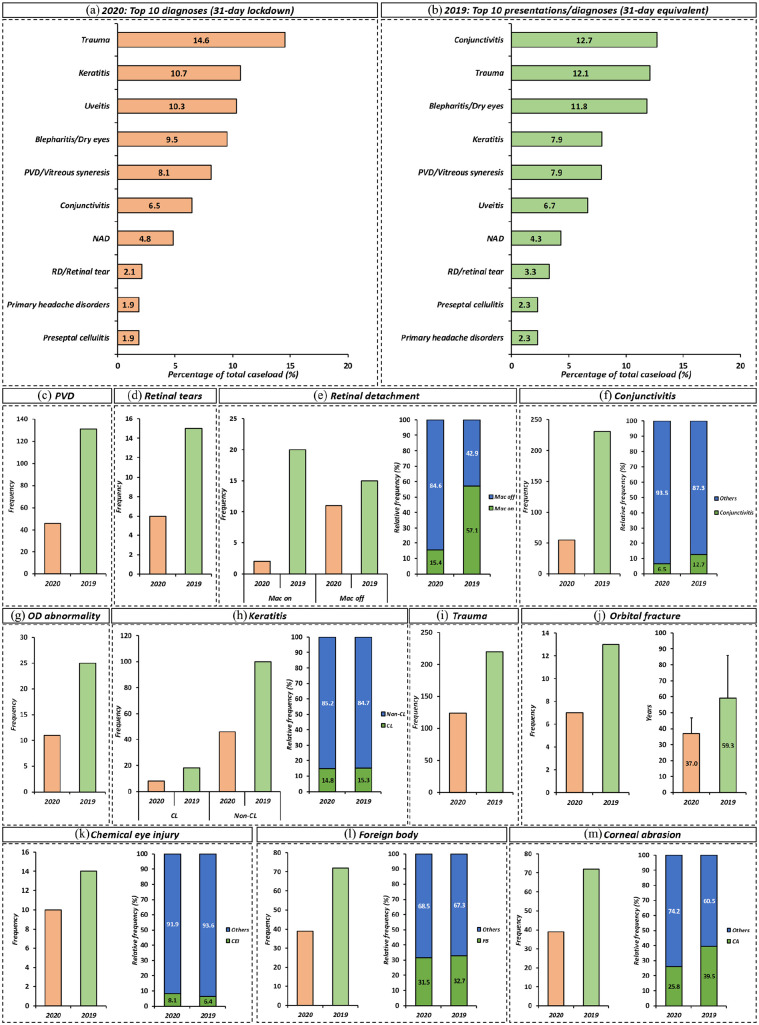
(a) Top 10 diagnoses presenting to the emergency eye department during COVID-19 related pandemic lockdown in 2020, (b) compared to equivalent period in 2019, (c) among vitreoretinal pathologies a reduced number of cases were seen for posterior vitreous detachments (PVD), (d) retinal tears, (e) and retinal detachment during lockdown. The proportion of macula off retinal detachments was significantly higher during lockdown (e), (f) reduced numbers of conjunctivitis, (g) optic disc (OD) abnormalities, (h) and keratitis was seen during 2020. The proportion of conjunctivitis (proportion of total caseload) was significantly reduced between 2020 and 2019 (f), (i) the number of contact lens (CL) related keratits was reduced in 2020, however as a proportion (of total keratitis caseload) this was not significantly different between 2020 and 2019. Reduced number of cases of ocular trauma were seen in 2020 compared to 2019, (j) this included orbital fracture, (k) chemical eye injury (CEI), (l) foreign body (FB) and (m) corneal abrasion (CA). Bar charts with mean and standard deviation of ages of patients with orbital fracture are shown (j). The proportions in relation to total trauma caseload are shown (k, l, m). RD: retinal detachment; NAD: no abnormality detected.

Plots of the top 10 diagnoses as a percentage of total caseload seen in EED during SP1 and SP2 are shown in Figure 3. During SP1, the top three diagnostic categories were trauma-related (percentage of total caseload: 14.6%), keratitis (10.7%) and uveitis (10.3%) which accounted for over 30% of the total caseload seen in EED. Contrastingly, in SP2 the top three diagnostic categories were: conjunctivitis (12.7%), trauma-related (12.1%) and blepharitis or dry eyes (11.8%), thus again accounting for over 30% of total caseload during SP2.

### Vitreoretinal

We observed a 60% reduction in the number of retinal tears presenting to EED during SP1 (*n* = 6) compared to SP2 (*n* = 15) (Figure 3(d)). Similarly, we had a 64.9% reduction in posterior vitreous detachment (PVD) presenting to EED between SP1 (*n* = 46) and SP2 (*n* = 131) (Figure 3(c)). We observed a 65.7% reduction in number of retinal detachments between SP1 and SP2. There was a significantly (*p* = 0.0099) higher proportion of macula-off retinal detachments (84.6% of all retinal detachments) during SP1 when compared to SP2 (42.9%) (Figure 3(e)).

### Neuro-ophthalmology

There was a 56% reduction in optic disc abnormalities (papilloedema, optic disc swelling, pseudopapilloedema and optic atrophy) presenting to EED during SP1 (*n* = 11) compared to SP2 (*n* = 25) (Figure 3(f)). However, we did not find a significant difference in the overall proportion (*p* > 0.05) of cases between SP1 and SP2.

### Anterior segment

We observed a significant difference (*p* < 0.0001) in the proportion of cases of conjunctivitis between SP1 (6.5% of total caseload) and SP2 (12.7%) (Figure 3(f)).

The proportion of cases of blepharitis and/or dry eye were reduced in SP1 (9.5%) compared to SP2 (11.8%), but overall the difference in proportions were not significant (*p* > 0.05).

In SP1, Keratitis (marginal, suspected infective (Contact lens (CL) related vs non-CL related)) had a significantly (*p* = 0.019) higher proportion (10.7%) of the total caseload compared to SP2 (7.9%). The number of CL-related keratitis was less in SP1 (*n* = 8) compared to SP2 (*n* = 18) (Figure 3(h)), however there was no significant difference (*p* > 0.05) in the proportions of CL-related keratitis between SP1 and SP2.

### Trauma

There was a 43.6% reduction in the number of trauma-related presentations/diagnoses in SP1 (*n* = 124) when compared to SP2 (*n* = 220) (Figure 3(i)). The number of corneal foreign bodies (FB) (*n* = 39) and chemical eye injuries (CEI) (*n* = 10) were lower in SP1 when compared to SP2 (*n* = 72 for corneal foreign body; *n* = 14 for chemical eye injuries) (Figure 3(k) and (l)). However, the proportions FB and CEI (as a proportion of total trauma-related diagnoses) were not significantly different (*p* > 0.05) between SP1 and SP2. The number of corneal abrasions was reduced in SP1 (*n* = 32) when compared to SP2 (*n* = 87). The proportion of corneal abrasions (as a proportion of total trauma-related diagnoses) was significantly reduced (*p* = 0.010) between SP1 (25.8%) and SP2 (39.5%) (Figure 3(m)).

There was a 46% reduction in the number of orbital fractures between SP1 (n = 7) and SP2 (*n* = 13) (Figure 3(j)). We observe orbital fractures in younger patients during SP1 (mean age ± SD = 37.0 ± 10.4 year) compared to SP2 (mean age ± SD = 59.3 ± 27.7 years) however this was not statistically significant (*p* = 0.057).

## Discussion

In this study we highlight the changing trends in ocular pathologies presenting to an eye emergency department during COVID-19 pandemic related lockdown. As anticipated, the overall number of EED attendances have approximately halved during the pandemic lockdown. This is consistent with the drop in the number of patients presenting to the emergency departments, in general, across England based on data from EDISS.^9^ The reasons for the overall reduction in attendance may be multifactorial including change in health seeking behaviour due to the lockdown, difficulty organising travel arrangements, fear of being infected during travel or hospital attendance. Previous community services led by consultants were stopped during the lockdown, however new initiatives such as the COVID-19 Urgent Eye Services (CUES) were introduced in the community which may influence patient attendance at a tertiary eye unit. Disease specific trends are discussed below. As expected, the ‘less serious’ conditions such as conjunctivitis, blepharitis, dry eyes were seen less frequently during SP1 compared to SP2. Conversely, though the number of cases of more serious pathologies (e.g. keratitis, uveitis, trauma-related) were lower in SP1, the proportion of these conditions of the total caseload seen were higher. Moreover, they represented the top three diagnoses presenting to our EED. We anticipated the lockdown measures, particularly the ‘shielding’ measures for those above the age of 70, would have resulted in a younger demographic presenting to EED. However, surprisingly, the average age of patients attending EED was not significantly different between SP1 and SP2.

The reduction in cases of conjunctivitis in SP1 presenting to EED could be multifactorial. Measures to stop spread of COVID-19 such as awareness of hand hygiene practices, social distancing measures and school closures could have an indirect role in reducing spread of other contagious diseases including viral and bacterial conjunctivitis. Moreover, national social media awareness campaigns advising not to attend eye casualty for conjunctivitis and possible reluctance to attend hospitals due to risk of acquiring COVID-19 infection could also be contributory. Similarly, there was decrease in the number of cases of keratitis, including contact-lens related keratitis during SP1. This again could be attributed to behavioural changes (e.g. improved hand hygiene practices) during lockdown and potentially reduced use of contact lenses. General practitioners have also been providing telephone and video consultation thus a proportion of conjunctivitis or ‘red eyes’ could have been managed in the community. However, this requires further study.

We identified a lower number of PVDs and retinal tears attending EED during SP1. Although the total number of retinal detachments were reduced during SP1, we identified a significantly higher proportion of macula-off retinal detachments during SP1 when compared to SP2. This is a particularly concerning statistic since it could imply that the impact of COVID-19 related lockdown has potentially delayed individuals with retinal tears and macula-on retinal detachments seeking medical help and thus progressed to macula-off retinal detachments. Additionally, the lack of access to optometrists could be contributory to less retinal tears picked up in the community and subsequently higher proportion of macula-off retinal detachments.

We found a lower number of optic disc pathologies presenting to EED during the COVID-19 pandemic. Consistent with the trends seen with retinal tears, we hypothesize this could also be due to the lack of access to community optometrists who often pick up a significant proportion of optic disc abnormalities during routine sight tests.

Pellegrini et al. reported a 68.4% reduction in ocular trauma, across different types of injuries, during the COVID-19 pandemic.^10^ In our study, we find that there was a 53.1% drop in cases of ocular trauma. We also report a reduced number of orbital fractures during SP1 when compared to SP2. Interestingly, we find the orbital fractures were in a younger demographic during the COVID-19 pandemic although not statistically significant. Overall this trend is likely to be related to a lockdown resulting in less outdoor activities.^10^ The effects of ‘shielding’ in the older population may explain the differences in the ages of patients diagnosed with orbital fractures during the COVID-19 pandemic.

Data from other specialties have also highlighted a concerning trend of patient mortality and morbidity unrelated directly to COVID-19.^9,11^ This is partly hypothesized to arise from delayed health seeking behaviour due to fear of contracting the virus by hospital attendance. Our data suggests that this extends to ophthalmology and raises concerns about increased morbidity likely to result from ocular pathology unrelated directly to COVID-19 (e.g. macula-off retinal detachments). Risk perception can influence behaviours.^12^ The perceived risk from subtle or less debilitating symptoms (e.g. flashes and floaters compared to significant ocular pain) paradoxically may not necessarily correlate with actual risk. Therefore, it is important for rapid data collection and sharing of results to ensure effective collaboration between clinicians, public health bodies and journalists^12^ thus informing the public to recognise symptoms that could potentially lead to sight loss. Going forward, ophthalmology services may need to prepare for a second ‘pandemic’ to cope with a likely rise in EED attendances and complications from delayed presentation.
